# Advancing a Non-Contact Structural and Prognostic Health Assessment of Large Critical Structures

**DOI:** 10.3390/s24113297

**Published:** 2024-05-22

**Authors:** Wing Kong Chiu, Thomas Kuen, Benjamin Steven Vien, Hugh Aitken, Louis Raymond Francis Rose, Matthias Buderath

**Affiliations:** 1 Department of Mechanical and Aerospace Engineering, Monash University, Wellington Rd, Clayton, VIC 3800, Australia; wing.kong.chiu@monash.edu; 2 Melbourne Water Corporation, 990 La Trobe Street, Docklands, VIC 3008, Australia; thomas.kuen@melbournewater.com.au (T.K.); hugh.aitken@melbournewater.com.au (H.A.); 3 Defence Science & Technology Group, Fishermans Bend, VIC 3207, Australia; francis.rose@defence.gov.au; 4 Airbus Defence and Space, Willy-Messerschmitt-Straße 1, 82024 Taufkirchen, Germany; matthias.buderath@airbus.com

**Keywords:** unmanned aerial vehicle, photogrammetry, structural health monitoring, prognostics, diagnostics, large critical structures, non-contact assessment, digital twin

## Abstract

This paper presents an overview of integrating new research outcomes into the development of a structural health monitoring strategy for the floating cover at the Western Treatment Plant (WTP) in Melbourne, Australia. The size of this floating cover, which covers an area of approximately 470 m × 200 m, combined with the hazardous environment and its exposure to extreme weather conditions, only allows for monitoring techniques based on remote sensing. The floating cover is deformed by the accumulation of sewage matter beneath it. Our research has shown that the only reliable data for constructing a predictive model to support the structural health monitoring of this critical asset is obtained directly from the actual floating cover at the sewage treatment plant. Our recent research outcomes lead us towards conceptualising an advanced engineering analysis tool designed to support the future creation of a digital twin for the floating cover at the WTP. Foundational work demonstrates the effectiveness of an unmanned aerial vehicle (UAV)-based photogrammetry methodology in generating a digital elevation model of the large floating cover. A substantial set of data has been acquired through regular UAV flights, presenting opportunities to leverage this information for a deeper understanding of the interactions between operational conditions and the structural response of the floating cover. This paper discusses the current findings and their implications, clarifying how these outcomes contribute to the ongoing development of an advanced digital twin for the floating cover.

## 1. Introduction

**Figure 1:**
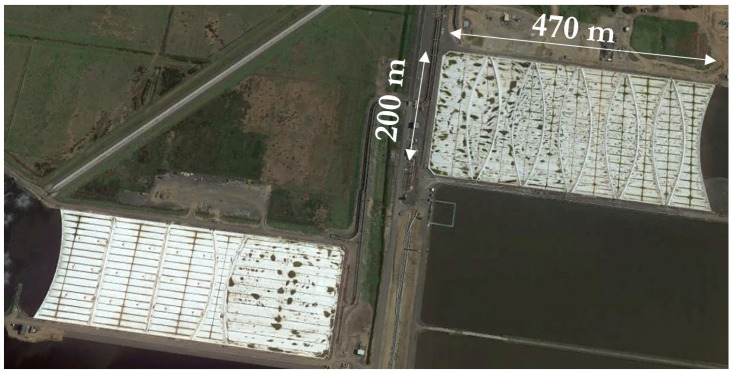
Anaerobic lagoons at the WTP.

Large membrane-like covers perform various environmentally sensitive roles, including (i) serving as floating covers for clean water reservoirs to mitigate evaporation and contamination; (ii) acting as liners for landfills to prevent the leakage of hazardous chemicals or substances; (iii) being utilised in mining applications such as heap leaching, salt evaporation ponds, and tailings impoundment [1]; and (iv) functioning as floating covers for anaerobic lagoons in wastewater treatment plants. Floating covers are vital assets at Melbourne Water’s Western Treatment Plant (WTP) in Werribee, Victoria. These covers typically span an area of 470 m × 200 m (refer to Figure 1), and are made from high-density polyethylene (HDPE). Known for its exceptional durability, HDPE for geomembranes has demonstrated, through accelerated testing, a service life exceeding 300 years at 20 °C and over 45 years at 40 °C [2,3]. As a result, well-designed HDPE geomembranes are anticipated to provide long-lasting performance without significant issues. These covers represent high-value assets, with construction and installation costs of tens of millions of dollars.

All sewage inflow remains unscreened and initially passes through the anaerobic section of the lagoon. The floating cover plays a crucial role in environmental conservation by capturing biogas generated during the anaerobic digestion of raw sewage. This biogas is converted into renewable electricity, surpassing the plant’s energy consumption needs. Thus, the floating cover emerges as a vital asset for renewable energy generation, contributing a significant role in environmental, social, and economic savings by preventing the escape of odours and biogas into the atmosphere.

However, accurately assessing the real-life mechanical performance of HDPE poses challenges [4]. The entry of untreated wastewater into the anaerobic lagoons leads to the formation and accumulation of solidified matter, or scum, underneath the covers, forming large volumes known as scumbergs. These scumbergs exert pressure against the covers, causing deformation with a vertical-length scale of around one meter (uplift) and several meters laterally. Hydraulic loading from the inflow of sewage can cause lateral displacement of the scumbergs, resulting in excessive displacement and mechanical stress on the covers, particularly in regions near the welded joints (refer also to [4]). This issue could disrupt biogas collection channels integrated within the cover, potentially reducing the efficiency of renewable energy collection. Therefore, managing and maintaining this critical asset for safe and efficient functionality is crucial.

Enhancing the ability to assess integrity efficiently will improve current practices, which rely on subjective and time-consuming visual walk-around inspections. Furthermore, the current inspection process lacks advanced warning of potential failures or clear indications of distress in the covers. Additionally, effective integrity management and maintenance of the floating cover’s integrity are essential for planning future cover replacement programs, potentially delaying such replacement and leading to significant financial savings for the asset operators. Recognising the importance and impact of such covers, the research team at Monash University seized the opportunity to work with Melbourne Water and investigate the application of recent advancements in structural health monitoring (SHM) technologies. The aim is to incorporate these innovations into pioneering engineering and maintenance “products”, ensuring the safe and efficient operation of this critical, valuable, and sizable infrastructure.

Remote sensing technology has become indispensable in today’s world, offering a safe and efficient means to collect crucial data, thus providing invaluable insight that informs decision-making across various disciplines [5,6,7,8,9,10,11]. An exemplar of this is the recent COVID-19 pandemic, underscoring the need for remote sensing for inspections or monitoring. For instance, a case study [12] used imaging techniques mounted on a robotic platform to monitor the patient’s temperature and respiratory rate. This non-contact measurement approach significantly reduces the risk to clinicians and prevents disease transmission. Mounting of sensors on UAVs offers an attractive alternative. Indeed, UAV-based structural health assessment strategies are widely reported, especially for inspecting and monitoring large structures or facilities. Several real-life case studies of these works include those reported in [6,7,8,9,10,11,13]. Furthermore, the study by Tsaimou et al. [14] resembles the work presented in this paper. The acquisition and manipulation of digital elevation models (DEMs) and mapping regions of distress were integral to their new condition assessment of port infrastructure. They used a UAV-enabled strategy to inspect the concrete surfaces and rubble mound structures. The ability to cover a large area is a testament to the efficiency of this technique. One significant advantage of a UAV-enabled inspection strategy is the ability to access hard-to-reach areas within structures and incorporate various remote sensing devices onboard [7]. Combining thermal images with optical images from UAVs significantly improves this remote sensing capability’s ability to detect features that one would miss when using only optical techniques. The most striking aspect of UAV-based methodologies is that these techniques integrate with analytical response models, which greatly enhance the capabilities of structural health assessment [10]. The UAV served as a remote sensing device to acquire data that were incorporated into a finite element (FE)-based damage model for damage assessment.

For Melbourne Water’s floating covers, only remote sensing-based monitoring techniques are suitable for the cover’s large size, the potentially explosive and hazardous environment due to biogas, and exposure to extreme weather conditions. Additionally, the science behind the formation and development of scumbergs from raw sewage beneath the cover remains an ongoing area of research. In addition, the scale of the formation of this matter and the hydrodynamics of the continual sewage flow into the lagoon defy conventional laboratory-based analysis tools for predicting scumberg formation. Our research has shown that the most reliable data for constructing a predictive model to support the structural health monitoring of this critical asset come directly from the actual floating cover at the sewage treatment plant. This paper presents a real-life case study of using UAV-based remote sensing capability for the SHM of this large floating cover.

In this industry-collaborative project, recent case studies [10,11,14] have demonstrated the efficacies of UAV-based remote sensing capability. This paper presents the research team’s innovative use of UAVs, equipped with optical cameras and GPS tracking, to monitor floating covers at the WTP. This UAV-enabled remote sensing capability was used to acquire data on the deformation of the floating cover due to the accumulation of matter beneath it. These data were instrumental in developing a time-progressing model that describes the accumulation process in ways that the current chemical knowledge cannot predict. We aim to harness UAV technology as a means for remote data collection, serving as primary inputs into analytics for meticulous processing and analyses to yield essential parameters to monitor the responses of the asset for management and maintenance. In the case study presented in this paper, data acquired by the UAV were analysed with a series of algorithms to provide insights into the covers’ behaviour under various operational conditions. This data acquisition and processing method enables economical, safe, and effective terrain mapping and integrity assessments, which are pivotal for evaluating structural integrity through non-contact methods. The effectiveness of this approach is highlighted by regular UAV surveys that generate DEMs at various times and produce comprehensive datasets. These datasets have been instrumental in improving the management and maintenance of the covers, including monitoring the formation of scum and scumbergs beneath them. Furthermore, this paper explores the potential for developing advanced diagnostic and prognostic tools to optimise this vital asset’s upkeep. Inspired by practices and the conceptual framework used in the aerospace industry, the aim is to establish an exemplary case study for developing a digital twin, ultimately revolutionising the approach to managing and maintaining floating covers.

## 2. Research Methodology

This paper aims to assemble the research outcomes from our focused studies to deliver a UAV-enabled remote-sensing case study on the SHM of a large floating cover. The nature of the problem addressed in this paper and the cover’s size preclude using small laboratory-scale experiments to develop monitoring strategies. The research team was required to conduct full-scale field data collection over several months to validate the floating cover’s DEM. These DEMs were then used to provide a variety of parameters to predict (a) the depth of matter (i.e., scum) accumulated under the floating cover, (b) the hardness of the scum formed under the cover, and (c) the gross movement of the cover, and (d) to estimate the strain on the cover. Wherever possible, these derived quantities were correlated with manual field data collected by the operators at the WTP. The work by [15] is an excellent example of the integration of “smart sensors” into smart grids (i.e., a form of smart structure), providing incisive insights on how smart sensors not only define the system’s intelligence but also emphasise the significance of interoperability among them.

The ability to determine the depth and hardness of the matter accumulated has direct operational implications for cover maintenance. These two parameters affect the cover’s ability to perform as a biogas harvester. Furthermore, determining the gross movement of the cover provides essential information to the operator of the WTP regarding the impact of operational decisions on the cover’s response and its structural integrity.

The results presented below demonstrate the effectiveness of this UAV-enabled remote sensing capability in delivering functional parameters for managing the floating cover. Any activity to remove the scum material formed beneath the floating cover would cost several millions of dollars in actual capital works, loss of electrical power generation from the inability to harness the biogas, and the release of the ozone-damaging gas into the environment. Therefore, integrating the research outcomes from a series of individual projects into SHM capability demonstrates the translation of research outcomes into a real-life case study. Furthermore, this integration supports the proposed use of these outcomes towards a knowledge-driven maintenance and management strategy for this critical infrastructure.

There is significant interest in digital twin technology in both industry and academia [16,17,18,19], as well as in the water industry [17,20,21]. Digital twins can facilitate a transition towards proactive management. In this management style, various processes and assets are strategically operated and maintained to preemptively address disturbances and other potential issues, thereby preventing significant detrimental effects on performance [21]. A case study [19] on a digital twin developed for the Singapore PUB Changi Water Reclamation Plant not only provided a 5-day hourly forecast and enabled operations in a safe environment under extreme conditions (i.e., emergency shutdowns, high flows, and equipment failure) but also enabled a more proactive operational mode through the use of information provided by the digital twin. Nevertheless, numerous studies indicate the necessity and importance of a framework and well-defined architecture tailored to the specific system for transitioning towards digital twins [16,17,18].

In SHM, integrating digital twins offers a powerful tool for predictive maintenance and is increasingly integrated into modern engineering practices [22,23,24]. Essentially, SHM systems provide the necessary data that feed into the digital twin, allowing for the real-time monitoring and assessment of structural integrity. The combination of SHM and digital twins enhances predictive maintenance capabilities by enabling decision-makers to visualise the impact of potential decisions and implement proactive strategies for maintenance and repair. This synergy not only improves the safety and longevity of structures but also optimises operational efficiency and reduces unnecessary expenditure by allowing for the precise timing of maintenance and repairs before significant damage occurs [22].

Buderath’s gap analysis work for the Aerospace Industry Steering Committee on Structural Health Monitoring, SAE International (AIR), expounded on the various stages of development of SHM in aerospace asset monitoring and assessment. Table 1 shows the interpretation and translation of these concepts to the floating cover. This translation will involve gap analysis to improve the end-to-end process in data collection and processing, asset state characterisation, and structural and performance assessment. We seek to use Buderath’s work to frame the progression and development of our SHM methodology for the floating cover at the Western Treatment Plant.

**Figure 2:**
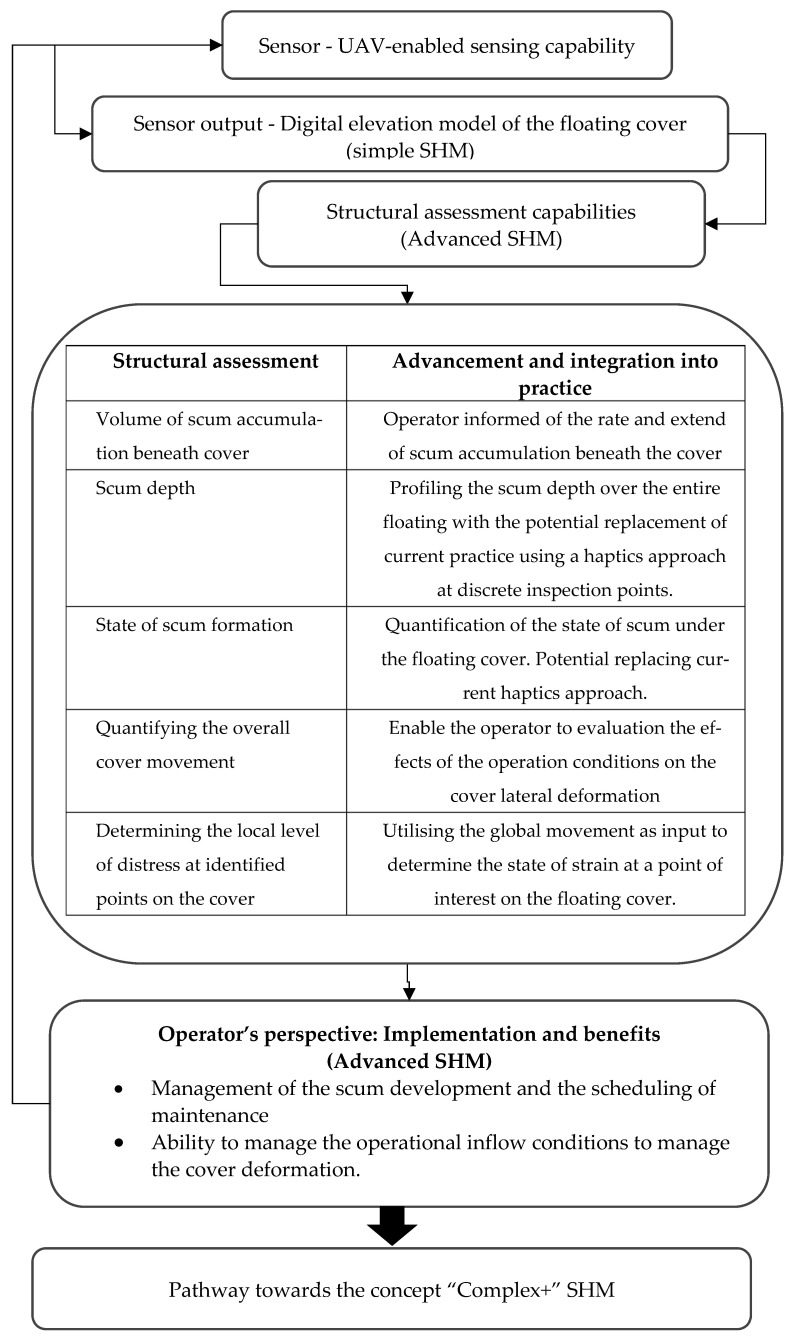
Schematic of SHM progressing.

The research methodology for transitioning through the various stages of development of the SHM methodology for the floating cover is illustrated in Figure 2. The UAV serves as the primary sensor for this monitoring procedure, delivering the DEM of the floating cover, which is the fundamental outcome of “simple” SHM. The digital information contained within the DEM was curated to provide parameters supporting our “advanced” SHM, enabling the monitoring and assessment of the floating cover’s response to changing operational input conditions. Here, we will be able to diagnose the extent of scum accumulation and also track its progression under consistent conditions (i.e., prognostics). The work presented in this paper then describes our pathway towards a “complex” and “complex+” SHM methodology, incorporating the potential of a digital twin for the floating cover.

## 3. Datasets Acquired from the UAV-Enabled Photogrammetry

Data collection, processing, enhanced diagnostics, and prognostics are the fundamental engineering functions that define structural health assessment capability. The UAV-enabled photogrammetry methodology acquires datasets to characterise the floating cover response, i.e., changes in elevation and volume [25,26,27,28], which has provided further insights for scheduling its maintenance.

The summary below provides an overview of our WTP work’s UAV-based data collection capability. It also outlines a series of data processing conducted to provide an understanding of this asset’s response to operating conditions over time. These works are essential building blocks towards diagnostic and prognostic capability. It is also important to note that this development process is industry agnostic, with the floating cover at WTP being an excellent case study.

This capability currently generates advisories to assist with the management and maintenance of the asset. Due to the voluminous data, a significant amount of preprocessing (i.e., resampling, reducing dimensionality, etc.) needs to be performed to ensure the data are of “high quality,” such that meaningful data are retained and the others are redundant, hence removed for more expedited analysis, reporting, and modelling. This work also underscores the need to develop helpful analysis tools to translate the large datasets to derive appropriate engineering quantities to understand the response of the floating cover. These tools will be the foundation of the engineering “products” for the floating cover management and maintenance team’s consumption for generating advisories, maintenance planning, and future life extensions.

### Digital Elevation Model

**Figure 3:**
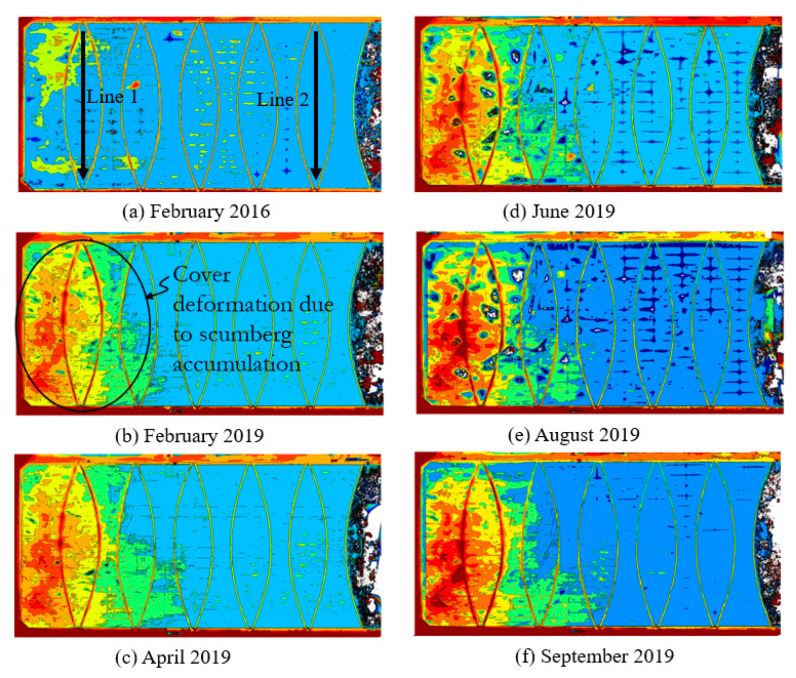
Floating cover DEM [28,29] on (**a**) February 2016, (**b**) February 2019, (**c**) April 2019, (**d**) June 2019, (**e**) August 2019 and (**f**) September 2019.

The UAV-enabled photogrammetry assessment technique was used to produce the floating cover’s DEM. These DEMs show the uplift of the HDPE cover due to the accumulation of scumbergs [25,26,27,28]. The work presented in [28] highlighted the transition of the exploratory tool to engineering practice. Figure 3a–f show the time progression of the vertical displacement of the floating cover created from the optical images acquired by the UAV over the floating cover at the WTP. The digital representation of the floating cover helps determine how the floating cover is being deformed and strained by the accumulation of scumbergs beneath it. On their own, these DEMs provide a visual perspective of the response of the floating cover. Furthermore, a 3D-printed solid model of this floating cover would provide a tangible and detailed representation, making it easier for engineers to visualise its impact.

**Figure 4:**
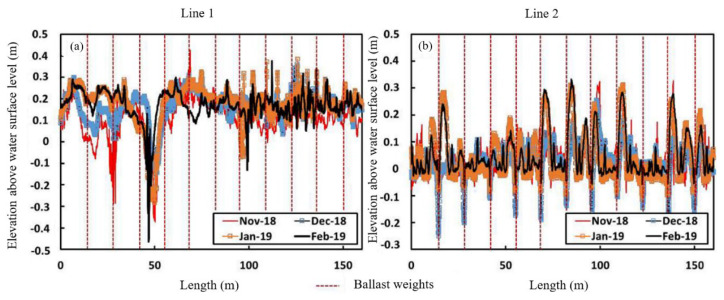
Cover vertical displacement along: (**a**) Line 1 and (**b**) Line 2 [29].

The DEMs show the cover deformation caused by the accumulation of scumbergs and the entrapment of biogas. The DEM analyses show the different length scales of the cover deformation caused by scumberg accumulation and biogas entrapment. Figure 4 shows the cover’s vertical displacement along Lines 1 and 2 (see Figure 3a), as described by Wong et al. [29]. The displacement along Line 1 showed an overall uplift of the cover. This displacement profile did not conform to the presence of the ballast installed along this location. However, the displacement profile along Line 2 appears constrained by the installed ballast. The entrapment of biogas in this region, where scumberg accumulation is insignificant, resulted in deformation with a length scale consistent with the ballast spacing. The corresponding length scale of the displacement profile due to scumberg accumulation is larger, as it lifts the ballast and the membrane together.

The DEM is the fundamental set of data from the UAV-enabled sensing methodology. These datasets provided the operators at the WTP with a basic set of results that allowed for them to appreciate the response of the floating cover with time. These DEMs provided pertinent diagnostic information that serves as a simple SHM tool.

The data embedded in this simple SHM tool can be curated and analysed to provide information that allows for the WTP operators to better understand the behavior and the response of the floating cover over a given period.

## 4. Gross Translational Movement of the Floating Cover

When formed, the scumberg can adhere to the underside of the HDPE cover. The continual inflow of raw sewage displaces these scumbergs, resulting in the lateral movement of the cover and causing it to wrinkle. The inflow conditions determine these wrinkling patterns and their progression. The data embedded in the DEMs of the floating cover can be analysed to diagnose this response, thereby enhancing the simple SHM tool to an advanced SHM tool, as illustrated in Figure 2. The analysis of these DEM data reveals the extent and progression of the cover wrinkles. These results provide useful information to inform the asset’s operator on the effects of the inflow conditions on the deformation of this critical asset [30].

We processed the DEM displacement data to highlight the cover’s wrinkling state. Since the wrinkles have a smaller length scale than the deformation caused by the accumulation of scumbergs and the entrapment of biogas, a highpass-filtered DEM provides a visual view of this wrinkling [28].

**Figure 5:**
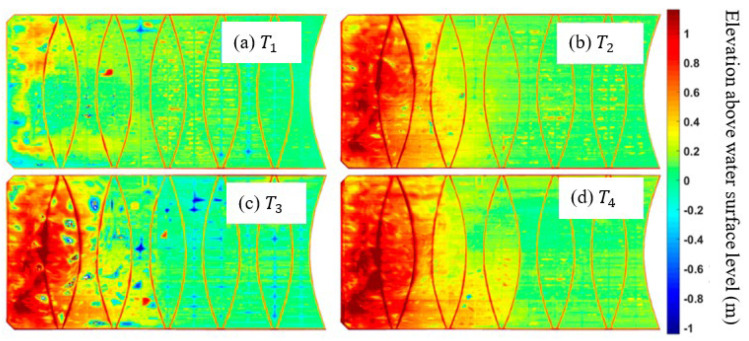
Floating cover DEM [30] at time (**a**) $T_{1}=\mathrm{the}\;\mathrm{year}\;2016$; (**b**) $T_{2}=T_{1}+3\;\mathrm{years}$; (**c**) $T_{3}=T_{1}+3.5\;\mathrm{years}$; and (**d**) $T_{4}=T_{1}+4\;\mathrm{years}$.

**Figure 6:**
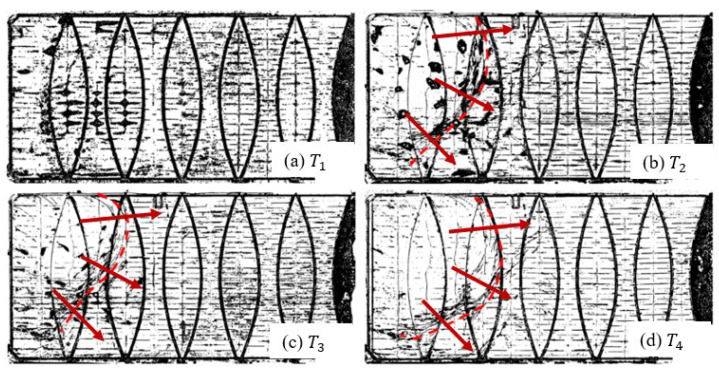
Highpass-filtered DEM showing the time progression of cover wrinkle (arrows indicate the direction of wrinkle propagation) [30] at time (**a**) $T_{1}=\mathrm{the}\;\mathrm{year}\;2016$; (**b**) $T_{2}=T_{1}+3\;\mathrm{years}$; (**c**) $T_{3}=T_{1}+3.5\;\mathrm{years}$; and (**d**) $T_{4}=T_{1}+4\;\mathrm{years}$.

Figure 5a–c show the DEMs acquired from 2016 to 2020 [30]. The time $T_{1}=\mathrm{the}\;\mathrm{year}\;2016$; $T_{2}=T_{1}+3\;\mathrm{years}$; $T_{3}=T_{1}+3.5\;\mathrm{years}$; and $T_{4}=T_{1}+4\;\mathrm{years}$. The highpass-filtered DEMs are shown in Figure 6a–c. The progression of the membrane wrinkling is evident, and more importantly, it provides a qualitative assessment of the cover’s response to the operational conditions. Delivering a quantitative evaluation of the cover’s movement will require data about the lateral displacements.

UAV-enabled photogrammetry captures a sequence of optical images of the floating cover, which are processed to generate an orthophoto. An opportunistic approach was experimented with to assess the capability of utilising these outputs to determine the floating cover’s global in-plane displacement. These in-plane displacements allow for us to quantify the gross movement of the floating cover. Wong et al. [30] introduced a FE-based formulation that leverages information from optical images to track the motion of known artifacts on the floating cover. The analysis of the principal components of the in-plane motion revealed regions of membrane stretch and wrinkle, which became dominant within 3 years.

**Figure 7:**
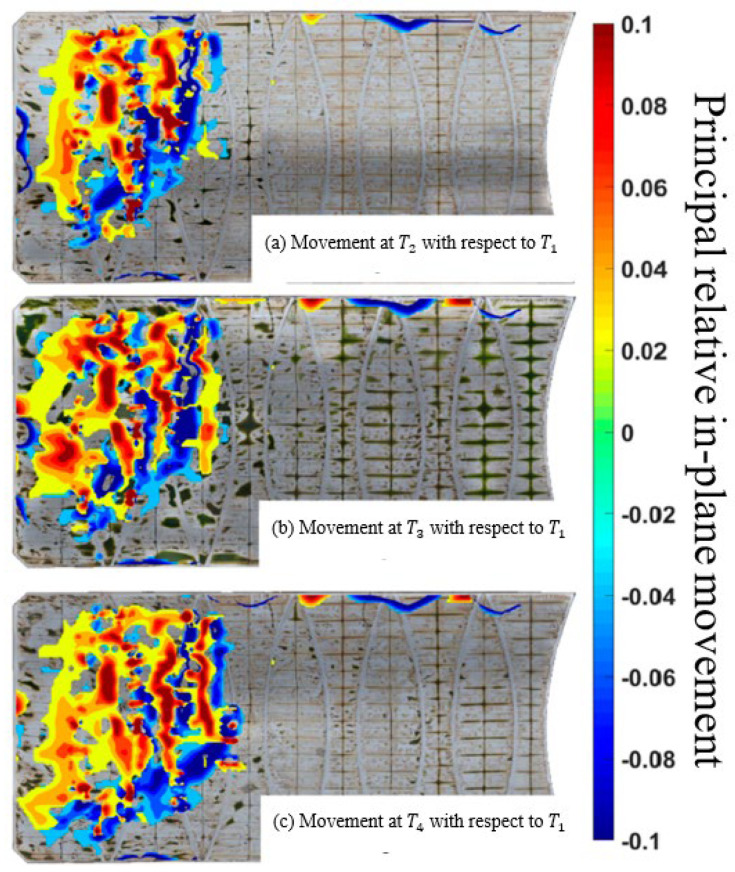
Principal relative in-plane movement [30] at time (**a**) $T_{2}=T_{1}+3\;\mathrm{years}$; (**b**) $T_{3}=T_{1}+3.5\;\mathrm{years}$; and (**c**) $T_{4}=T_{1}+4\;\mathrm{years}$ with respect to $T_{1}$.

The net movement of the floating cover at $T_{2}$, $T_{3}$, and $T_{4}$ is presented as the movement about its position at the time $T_{1}$ (i.e., Figure 7). As the state of strain of the floating cover is unknown during installation, estimating this global in-plane motion proves valuable in evaluating the impact of scum development and motion on the cover. The results depicted in Figure 7 show the maximum principal component of relative in-plane cover displacement predicted for the time intervals shown in Figure 7. The “blue” regions indicate the formation of wrinkles, while the “red” regions represent areas of global membrane stretch. This information allows for determining expected tensile loading regions and the extent of wrinkling.

The progression and geometry of the wrinkled region and the extent of membrane stretch are discernible in these results, providing a comprehensive view of the cover’s response. The gross movement of the floating cover shown in these results describes how the floating cover responds to the inflow conditions. These data will be integrated into our ongoing work to develop a global–local algorithm, offering local strain information for critical regions of the floating cover.

## 5. Predicting the State of Hardness of Scumbergs Formed Beneath the Floating Cover

The occurrence of scum and the formation of scumbergs are observed to disrupt the biogas channels integrated into the cover, potentially impacting the efficiency of collecting this valuable renewable energy source. Estimating the depth, extent, and state of scumberg formation and understanding its influence on biogas collection is crucial for assessing the performance of this asset. Presently, human operators conduct field measurements by walking and working on the floating cover to qualitatively assess the hardness of the scum underneath using scum hardness levels categorisation (H—hard, MH—medium hard, M—medium, F—fluffy or soft, scum, and W—watery). Experienced operators at the WTP perform this haptic assessment, which may various among different operators as it is subjective and dependent on the individual operator.

Observations suggest that the harder scum is more buoyant than softer ones. The buoyancies of scum led to the deformation of the floating cover that the DEMs characterise. The haptics assessment of the state of scum that has been accumulated under the cover resembles the shape of the DEMs. A machine-learning capability is developed to convert the DEMs into regions of different scum state. The haptics assessment served as validating data for this work, as described by Wong et al. [31].

**Figure 8:**
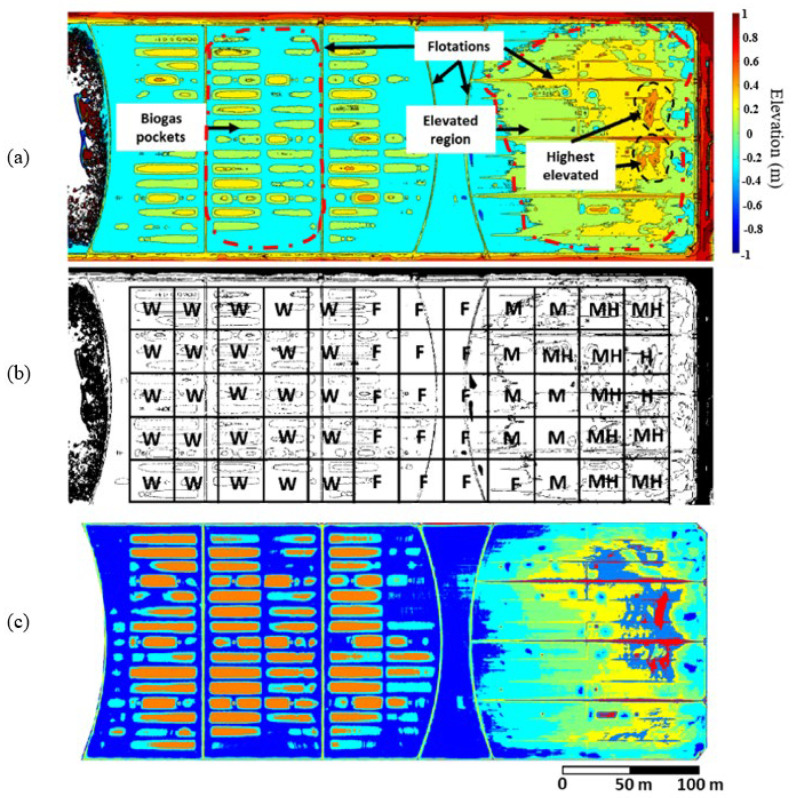
(**a**) DEM of the cover, (**b**) qualitative scum hardness survey (H-Hard, MH-Medium Hard, M-Medium, F-Fluffy and W-Watery) via cover walk haptics inspection and (**c**) k-means based image segmentation on DEM with 7 clusters [31].

Figure 8a shows the DEM of the scanned cover at the lagoon, which represents the height of the cover above the water surface level. The constructed DEM reveals that the covers in the vicinity of the sewage inlet are significantly elevated, especially along the middle section, attributed to the accumulation of hard scum at the water surface level. Figure 8b presents a grid indicating the qualitative hardness of the scum, haptically determined by an operator while traversing the entire cover. We subject the DEMs to clustering analysis, a common unsupervised machine-learning technique, due to insufficient labelled data to train, thereby allowing the ML to determine the patterns within the dataset. This analysis identifies hidden patterns or groupings in datasets without labelled responses. Image segmentation applies k-means clustering to partition the data into ‘k’ distinct clusters based on the distance to the centroid of a cluster. A 7 k-means clustering is applied to cluster the five different levels of scum hardness based on the elevation of the cover and to identify the biogas pocket and flotation. Figure 8c shows the results from this clustering analysis. Nevertheless, the cluster regions aligned with the haptic assessment, suggesting a significant correlation with elevation.

Using the elevation of the cover above water level, the cluster coloured in red aligns well with the ‘H’ region, as indicated in Figure 8b. Additionally, the clusters for both ‘MH’ and ‘M’ regions can be easily identified, along with the biogas pockets. This clustering method encountered challenges distinguishing the border between the ‘F’ and ‘W’ regions because we only used the vertical displacement as the input. This difficulty can be attributed to a subtle difference in elevation between the ‘F’ and ‘W’ regions. Nevertheless, the DEM image segmentation method adequately outlines the area of the ‘M,’ ‘MH,’ and ‘H’ regions beneath the cover. This DEM-enabled prediction methodology relates the elevation with the state of scum hardness. It does not require a person to walk over the entire floating cover and is a safer inspection method than the current walk-around haptics inspection practice.

## 6. Predicting the Depth of Scumberg Formed Beneath the Floating Cover

In the work presented in [31], the total depth of the scum is measured at discrete access ports within the cover, necessitating personnel to traverse the cover for access. Using a long and rigid rod, they gradually insert it into the access ports. The depth is recorded when personnel perceive a change in “feel” during insertion. The depth where the transition from hard or semi-hard scum to liquid sewage occurs is assessed using haptics. It is understood that, with an experienced operator at the WTP, this haptics technique can determine the depth of the scum with a tolerance of 0.1 m. However, this method is very subjective and currently the only method of estimating scum depth. The level of risk associated with this measurement technique is also evident, as it requires a person to be physically present on the cover. Our attempts to develop a machine learning capability to convert the DEMs into regions of different scum states led us to work on predicting the depth of the scum formed under the floating cover from its vertical displacement, as reported in Wong et al. [31].

**Figure 9:**
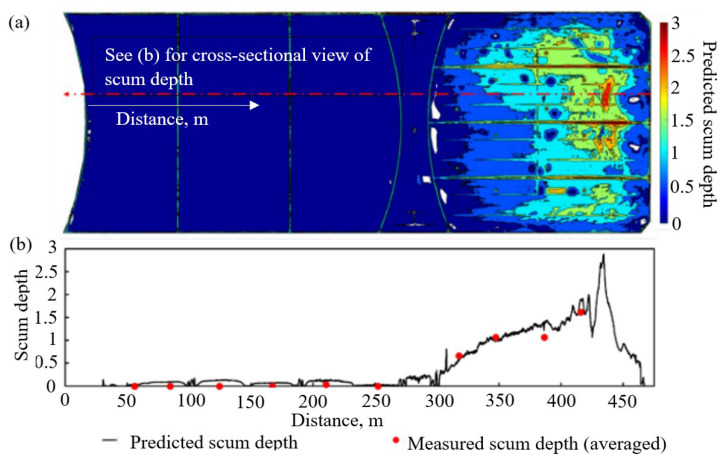
(**a**) Contour plot for the predicted scum depth obtained from filtered DEM and (**b**) cross-sectional view of scum depth along the highlighted line in (**a**) [31].

Figure 9 illustrates the correlation of the DEM at each access port with the corresponding total scum depth. A best-fit line in Figure 9b exhibits a 0.78 “goodness of fit”. For scum depth prediction, it is assumed that the elevation of the cover above the water level maintains a linear relationship with the scum depth. Figure 9a presents a contour plot of the predicted scum depth. A cross-sectional view of the expected scum depth is compared with the averaged scum depth obtained from physical measurements in Figure 9b, which indicates good agreement between the predicted and actual scum depth (averaged). Deducing the scum depth from the DEM will provide a continuous measurement over the entire cover, as opposed to the current measurement taken at discrete locations on the cover. It will also remove the risk associated with needing a person to physically walk on the cover to conduct these manual measurements. This DEM method of estimating the scum depth also removes the qualitative nature of the haptics associated with the current manual measurement. It must be noted that the work considered only the elevation of the floating cover, and other external factors, i.e., water level, environmental changes, etc., may influence and hence explain the deviation from this relationship. Nevertheless, we expect that the availability of more scum depth datasets will enhance this predictive model and estimate uncertainties arising from both intrinsic data and the model itself.

## 7. Strain Determination

The presence of scum and other loads results in the deformation (i.e., wrinkles and folds) of the floating cover, thereby exerting strains on the membrane. Given the size of the assets, the commercially available methods (i.e., digital image correlation) are inefficient for determining engineering quantitative methods. Initial work integrated photogrammetry-derived DEMs of an experimental deformed membrane using FE analysis by demonstrating the transfer of a DEM of a deformed membrane to FE to evaluate the strain measurements; however, only elevation information was considered to ensure convergence. Vien et al. [32] conducted FE and statistical analyses by employing cubic smoothing to effectively denoise the DEM and variational heteroscedastic Gaussian process regression (VHGP) to probabilistically reconstruct the loss of amplitude due to the smoothing process. Although it is shown that the predicted strain distribution has a profile similar to that of the optical fibres, significant uncertainty still exists. These uncertainties are attributed to several factors, including those arising from photogrammetry and preprocessing and postprocessing analyses. The uncertainties were especially noticeable in regions of high strain variance and noise, intensified by changes in the smoothing weighting parameter. In these regions, the average maximum change in strain was reduced by 25.7% to 68.6% for wrinkle deformations due to a three-order-of-magnitude difference in the smoothing cubic spline weighting parameter. In addition, inaccuracies in strain measurements were suspected to arise from using UAV photogrammetry in areas with steep slopes, where previous studies [32] have encountered difficulties reconstructing features with sharp corners and high-gradient slopes. However, enhancing the number of viewing angles may improve the accuracy of photogrammetry analyses. It was also noted that the feature identification algorithm artificially induced erroneous displacements, contributing further to the data’s unreliability and compounding the overall uncertainty. Nonetheless, ongoing work is focused on adopting learning models to capture robust strain for this large asset.

## 8. Towards a Complex+ Structural Health Monitoring Framework for Floating Cover Diagnostics and Prognostics

The floating cover is a high-value asset whose construction and installation costs tens of millions of dollars. This asset brings significant environmental, social, and economic benefits because it prevents odour and biogas from being released into the atmosphere. In addition, the ability to collect biogas makes this floating cover an important renewable energy-generating asset. In this respect, effective integrity management and maintenance of the floating cover will deliver the mentioned benefits, and these intelligent strategies are also crucial for planning future cover replacement programs.

As discussed above, our current UAV-enabled inspection methodology delivers large datasets to create a DEM of the floating cover. These datasets are processed and analysed to provide information for diagnosing the cover and its deformation state. The gap analysis work proposed by Buderath is an excellent platform for a systematic and logical process to transform our floating cover monitoring strategy towards the floating cover digital twin outlined in Table 1. The analyses described above show that we are on our way to achieving “Advanced” health integrity and assessment; accumulating the datasets will enable us to strive towards the Complex and Complex+ status.

Our individual findings have contributed to the SHM research field. The integration of these findings has led to the development of an integrated SHM methodology for the floating cover. With the data provided by the optical images from a UAV “sensor”, five important parameters were determined from the algorithms that quantify the performance of the large asset. As illustrated in Figure 2, this facilitated the development of a diagnostic and prognostic tool that exceeds the realm of research and is embraced by the practitioners at the WTP. Whilst in its infancy, these parameters have provided the asset operator with an understanding of the response of the floating cover to the operating conditions over time, making them useful diagnostic tools for the asset operator. Accumulating future datasets and their corresponding analyses will help develop and verify the prognostic capability. These will be the foundation of a “product” for managing this crucial asset.

One significant practical implication of this concept is defining the asset “maintenance-free” period from the outcomes derived from this work. By integrating the operational conditions with the structural conditions/health of the asset, the floating cover digital twin will enable us to determine the appropriate operating conditions under which the asset can operate that will ensure structural safety before the subsequent scheduled maintenance action.

Harnessing the biogas generated during the anaerobic reaction is one of the critical functions of the floating cover. Work is underway to acquire and understand the volume of the gas harness during a yearly cycle, as well as the operating conditions, including sewage inflows and maintenance actions performed [33,34]. This work describes a rapid training strategy that can predict biogas production more accurately when historical data include significant outliers. A trained model with fewer input variables via the feature selection technique based on the correlation coefficient yielded good performance given enough dataset training. The overall best performance model comprises the reduced input variables and data processed with z-score standardisation. This initial study provides a helpful guide for implementing machine learning techniques to develop more innovative structures and management towards Industry 4.0 concepts.

Integrating diagnostic capability with biogas production enables the asset operator to appreciate the effects of the floating cover’s structural conditions on the asset’s performance in biogas delivery during its service lifetime. These will be the critical functions of the floating cover digital twin. The objective is to facilitate safe biogas production while considering the floating cover’s structural integrity given a set of operating conditions. This capability will enable this asset to deliver financial benefits from biogas production. A predictive model will maximise biogas production by optimising the frequency of maintenance and downtime while ensuring asset integrity.

Consequently, it will reduce the risk and frequency of failures, thereby decreasing repair needs and effectively cutting costs with the new prognostic capabilities. As a result, this approach is expected to save the asset operator millions of dollars. This efficient integrity assessment “product” will assist and improve maintenance practices by providing data-based warnings of possible failures or clear indications of distress in the covers. It also promotes safety by reducing or eliminating the need for the visual walk-around inspection. This intelligent diagnostic and prognostic “product” will be helpful in life-extension decision-making with the potential of delaying its replacement. This model will enable the operator to consider changing the operating conditions for a set period to achieve safe biogas delivery and schedule the necessary maintenance action. In this respect, this “product” can potentially be used to optimise maintenance scheduling and reduce/eliminate unplanned maintenance/shutdowns. It will also help maintain target biogas production levels whilst ensuring asset integrity, therefore managing the asset risk profile. These will lead to an intrinsic cost reduction in the asset. The work by [15] provides a comprehensive overview of the required communication between the sensor–algorithm–floating cover, which is crucial for this “product.” This knowledge will also provide insight into this asset’s response to operational conditions and serve as guidelines for future cover design.

The immediate impact of this work is showcasing the transformation of the current manual maintenance and inspection of large assets operating in hazardous environments. In addition to using this new SHM capability, leading to a knowledge-driven maintenance and management strategy for this critical infrastructure, this remote assessment strategy will significantly enhance safety in this critical asset by allowing for human operators to remain safe from this hazardous environment.

## 9. Conclusions

This paper presents an overview of integrating new research outcomes into developing a structural health monitoring strategy for a large floating cover at the WTP in Melbourne, Australia. The size of this floating cover, which covers an area of approximately 470 m × 200 m; the hazardous environment; and its exposure to extreme weather conditions only allow for monitoring techniques based on remote sensing. This paper presents the assembly of the research outcomes from our focused studies to deliver a UAV-enabled remote sensing case study on the structural health monitoring of a large floating cover. This work demonstrates the derivation of five essential parameters determined from the algorithms that quantify the performance of the large asset. It also shows how these capabilities have facilitated the development of a diagnostic and prognostic tool that has exceeded the realm of research and is embraced by the practitioners at the Western Treatment Plant. Whilst in its infancy, these have provided the asset operator with an understanding of the response of the floating cover to the operating conditions over time, which is a useful diagnostic tool for the asset operator. One significant practical implication of this concept is defining the asset “maintenance-free” period from the outcomes derived from this work. By integrating the operational conditions with the structural conditions/health of the asset, this work paves the way for the future development of the floating cover digital twin, which will enable us to determine the appropriate operating conditions under which the asset can operate, ensuring structural safety before the subsequent scheduled maintenance action.

## Figures and Tables

**Table 1:** Relevance of SHM concepts to the floating cover.

Category of Health and Integrity Assessment	Capabilities	Relevance to Floating Cover
Simple	Diagnostic	Cover deformation; presence of defects as a function of time; leakage detection
Advanced	Diagnostic + prognostic	What are the implications of these measurands on Integrity of the cover Performance of the cover (methane harnessing and sewage processing) Maintenance actions
Complex	Diagnostic + prognostic + bidirectional communication (e.g., recalibration and reconfiguration)	Assessing the impact of maintenance actions on State of deformation of floating cover Performance of floating cover Calibration of the factor of safety in the cover Configuring the maintenance plans
Complex+	Diagnostic + prognostic + bidirectional communication + maintenance planning Definition of “maintenance credit”	Development of a digital twin capable of health and prognostic monitoring of this key asset Definition of maintenance-free concept for the floating cover

## Data Availability

Data are contained within the article.
